# Outcomes of parenteral nutrition in patients with advanced cancer and malignant bowel obstruction

**DOI:** 10.21203/rs.3.rs-3455273/v1

**Published:** 2023-11-14

**Authors:** David A. Velasquez, Ankit Dhiman, Colette Brottman, Oliver S. Eng, Emily Fenton, Jean Herlitz, Edward Lozano, Edwin McDonald, Valerie Reynolds, Elizabeth Wall, Jeffrey Whitridge, Carol Semrad, Kiran Turaga, Dejan Micic

**Affiliations:** University of Chicago

**Keywords:** Malignant bowel obstruction, parenteral nutrition, advanced cancer

## Abstract

**Background:**

Malignant bowel obstruction (MBO) affects 3–15% of all cancer patients. In patients with advanced cancer and inoperable MBO, the average survival varies between four to nine weeks. Parenteral nutrition (PN) may improve survival in specific patient populations with malignant bowel obstruction.

**Aims:**

This retrospective, single-center cohort study aimed to review individual patient outcomes on PN in the setting of advanced cancer with a diagnosis of MBO and identify clinical and laboratory markers predictive of short- and long-term survival to further highlight patients that would benefit from PN in the setting of an inoperable MBO.

**Results:**

In a retrospective analysis of 68 patients receiving PN for inoperable MBO, the median survival was 142 (IQR: 63.3–239.5) days. Patients experienced a median number of two hospital readmissions (range: 0–10) and spent a median of 29 days (range: 0–105) in the hospital after starting PN. Eighteen (26.5%) patients developed a catheter-related bloodstream infection (CRBSI). A diagnosis of appendiceal cancer was identified as a predictive marker of improved survival (HR: 0.53, 95% CI: 0.29–0.92, p = 0.023).

**Conclusions:**

The use of PN in the context of end-of-life cancer care is a practice that necessitates improvement. Recognizing the outcomes and patient experiences of PN utilization is essential to physicians and patients.

## Introduction

Malignant bowel obstructions (MBO), defined as bowel obstructions that inhibit physiological transit and digestion, are a common complication in patients with advanced stages of cancer (1). When unaddressed, MBO are often recurrent and can result in severe malnutrition with an impaired quality of life. In patients with advanced cancer and inoperable MBO, the average survival is approximately four to nine weeks (2). It is currently estimated that MBO affect 3–15% of all cancer patients; however, rates of MBO are higher in specific subsets of cancer, namely gastrointestinal and gynecologic cancers (2). For example, 10–28% of patients with metastatic colorectal cancer and up to 80% of patients with gynecologic cancer subtypes suffer from MBO in their disease course (2, 3).

As a clinical intervention, patients often receive intravenous parenteral nutrition (PN) in order to manage the progressive malnutrition from MBO (5). Intravenous nutrition includes the provision of macronutrients (carbohydrates, protein, and fat), micronutrients, electrolytes, and water necessary for survival. Thus, administering PN and bypassing the gastrointestinal tract may prolong survival and provide nutritional support to patients allowing for a continuation of cancer treatment, such as surgery or chemotherapy (6,7). However, the benefits of PN for oncologic patients with advanced MBO are not fully understood. Past studies have demonstrated that only about 30% of patients with a MBO surviving greater than three months benefit from PN with respect to improvements in quality of life (1). Furthermore, few studies have reported the overall survival of patients with advanced cancer and MBO based on different cancer subtypes or have identified predictive factors associated with increased survival (8, 9). The evidence for whether home PN (HPN) improves survival or quality of life in individuals with MBO is very uncertain due to the limited available evidence for both outcomes (10). Therefore, the effectiveness of the utilization of PN in patients with advanced cancer and MBO is therefore debatable and requires additional data supporting its use.

To expand on the current medical literature, our aim was to retrospectively review individual patient outcomes on PN in the setting of an advanced cancer with a diagnosis of a MBO at our institution. In addition, our goal was to identify individual predictors of patient survival at baseline and early in the course of PN therapy in order to identify patients that will most likely benefit from PN use with respect to overall survival.

## Methods

### Participants and study settings

A retrospective review was performed including all patients 18 years of age or older with a diagnosis of advanced cancer and an admission for a MBO at our institution between 1/1/2013 and 4/1/2021. Cases of MBO were identified using ICD-9 and ICD-10 codes as detailed in Supplementary Table 1 and separately reviewed based on clinical, radiographic, and histologic information.

**Figure 1:**
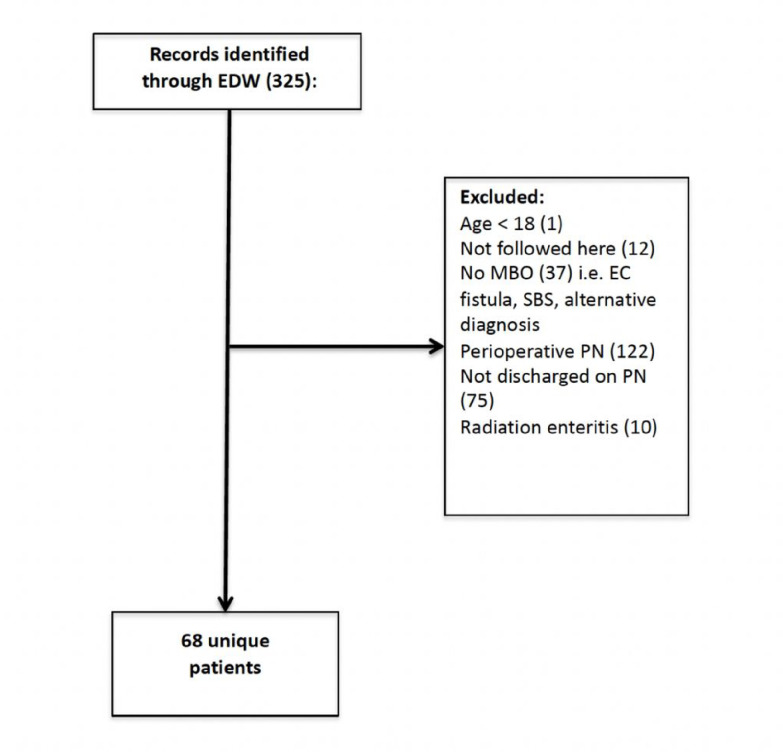
The patient selection process for the study. Comprehensive screening was performed 325 subjects identified via electronic medical record search. Exclusions included: age < 18 (n=1), treatment at another institution (n=12), receipt of an alternative diagnosis (n=47), receipt of perioperative (n=122) or temporary (n=75) parenteral nutrition (PN). A total of 68 patients were included in the final analysis.

Patients were excluded for age < 18 (n = 1), admissions for alternative diagnoses that were not for MBO (i.e., enterocutaneous fistula, short bowel syndrome, radiation enteritis; n = 47), admissions requiring only perioperative PN (n = 122), a discharge without PN (n = 75), and other (i.e., receiving care at another institution resulting in untraceable clinical data; n = 12) (Fig. 1).

Only patients discharged on PN for MBO were included in the analysis. This study was approved by the Institutional Review Board (IRB18–1505).

### Clinical characteristics

Baseline and outcome measures were collected from the electronic medical record system (EMR). Variables collected based on chart review included cancer characteristics (date of cancer diagnosis, site of the primary tumor, metastasis, peritoneal carcinomatosis), PN characteristics (date of initiation, type of catheter, duration of treatment, PN-associated complications), peritoneal cancer index (PCI), Eastern Cooperative Oncology Group (ECOG) performance status, prior admissions, readmissions, hospice enrollment, and history of chemotherapy, radiation therapy, and surgical procedures performed prior to and during the course of hospitalization. The following laboratory values were also recorded and followed from the date of PN initiation: weight, BMI, percent weight loss, C-reactive protein (CRP), lymphocyte count, hemoglobin, white blood cell count, alanine transaminase, renal function, serum albumin, and serum pre-albumin. Complications of a MBO and PN utilization included abdominal pain, catheter-related bloodstream infections (CRBSI), electrolyte disturbances following the initiation of PN, sepsis, and upper and lower extremity deep vein thromboses (DVTs). Complications were included from primary and secondary diagnoses within the reason for admission and discharge summaries of individual hospitalizations.

### Clinical outcomes

The primary outcome of interest was patient survival on PN calculated between the date of PN initiation to the date of death. Identifiable clinical risk factors were assessed as predictors of overall survival. Secondary outcomes of interest included identification of PN-related complications and number of hospital readmissions following PN initiation.

### Statistical analysis

The primary outcome of interest was overall survival, and the secondary outcome was the identification of predictors of overall survival. Demographic data and complications data are presented as mean ± SD or median (IQR). Kaplan-Meier curves were generated for time-to-event data, that is from the date of PN initiation until the date of death. A Cox regression model was performed to examine the effects of predictor variables, namely demographic features, disease type and baseline laboratory values, on the time to death. Associations are described as hazard ratios (HR) with 95% confidence intervals (CI). Univariate logistic regression was used to examined predictors of 30-day hospital readmission. A two-sided *P*-value ≤ 0.05 was considered statistically significant. Statistical analysis was conducted using JMP ^®^ 13.1.0 (SAS Institute, Inc., Cary, NC).

## Results

### Demographic characteristics

Three hundred twenty-five patients were initially identified through an electronic search of medical records, among which 68 with advanced cancer and a MBO were discharged on home PN between 1/2013 and 4/2021 and met the inclusion criteria (Fig. 1). The median patient age was 57.5 years (IQR: 50.3–66) and 38 (55.9%) were female. Appendiceal cancer was the most common malignancy (n = 24, 35.3%) followed by colorectal cancer (n = 15, 22.1%), gastric/gastroesophageal (n = 8, 11.8%) and gynecologic cancer (n = 7, 10.3%). Baseline demographic information, cancer characteristics, and laboratory values are listed in Table 1.

### Baseline clinical status

Forty-five (66.2%) patients had good to moderately impaired overall performance status (ECOG 1–2), and 14 (21.5%) were assessed as ECOG 3 (capable of only limited self-care, confined to bed or chair more than 50% of waking hours). One patient (1.5%) was characterized as ECOG 4 (completely disabled; cannot carry on any self-care; totally confined to bed or chair). Twenty-nine (46%) patients had a weight loss of greater than 10% in the six months before starting PN and 18 (25.7%) patients had a BMI of < 20.

### Survival analysis

**Figure 2:**
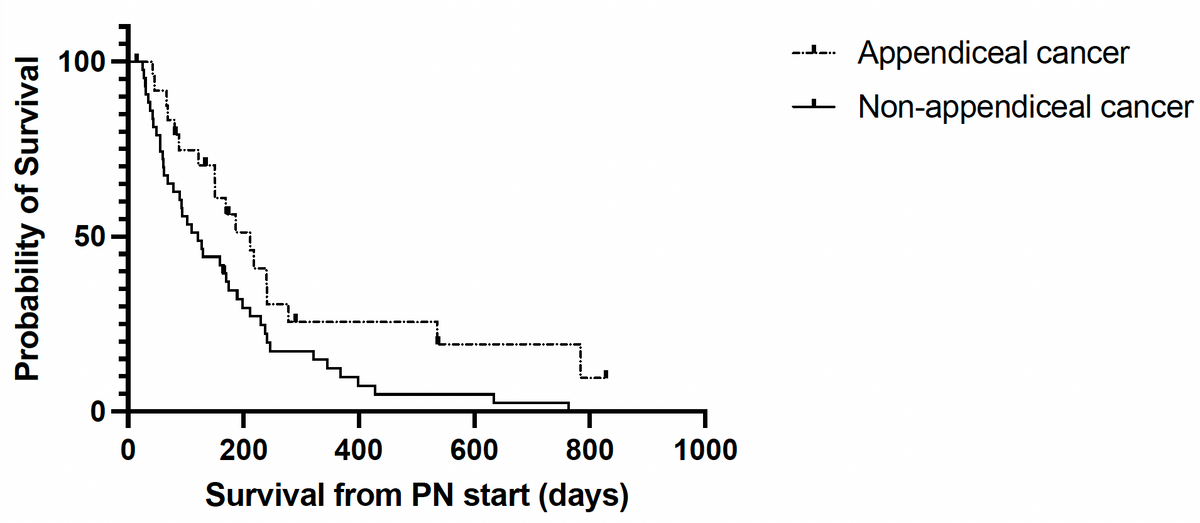
Survival analysis demonstrating prolonged survival among individuals with appendiceal cancer as opposed to non-appendiceal cancer (Hazard ratio: 0.53 (95% CI: 0.29–0.92, p = .02)).

The median overall survival time was 4.7 months (IQR: 2.1–8) from the time of PN initiation. Fifty-eight (86.6%) patients survived greater than 1 month, 38 (57.6%) survived greater than 3 months and 23 (35.9%) survived greater than six months. Patients with appendiceal cancer had statistically significant greater overall survival compared to patients with non-appendiceal cancer patients, demonstrating a reduced hazard ratio of 0.53 (95% CI: 0.29–0.92, p = .02) for death (Fig. 2).

No other baseline clinical predictors were significant to predict overall survival (Table 2).

When survival was analyzed as a categorical variable for predictors of 3 and 6-month survival, no additional predictors were significant (data not shown).

### Hospital readmission

The initial length of stay in the hospital for the MBO and initiation of PN was 15 days (IQR: 9–19). The median number of hospital readmissions and number of days spent in the hospital after starting PN were 2 readmissions (IQR: 1–3) and 29.5 days (IQR: 19.3–44), respectively (Table 3).

As a percent of survival time from the initiation of PN, patients spent 20.8% of their remaining time alive in the hospital.

### Predictors of hospital readmission

The overall 30-day readmission rate was 52.2% (35/67). Older patients were less likely to be readmitted to the hospital (p = 0.037). There was no significant difference in readmission rates within 30 days of starting PN between gender, race, BMI, weight, weight loss > 10%, initial albumin value, cancer type, or receipt of additional chemotherapy (p = NS), as shown in Table 4.

### Complications experienced while on parenteral nutrition

Multiple complications were experienced by patients on PN, shown in Table 3. Fifty-one (75%) patients were subsequently admitted to the hospital for abdominal pain and 25 (36.8%) patients had hospitalizations for sepsis/bacteremia. Specific to PN utilization, 18 (26.5%) had a catheter related bloodstream infection (CRSBI). Five (7.3%) patients developed an upper extremity and 7 (10.3%) developed lower extremity deep vein thromboses (DVT). Twenty-three (33.8%) patients developed ascites and twenty-two (32.4%) patients developed pleural effusion. Among individuals with a CRBSI, no difference in overall survival was identified (p = NS) but individuals with a CRBSI did have a greater number of total days spent in the hospital (47.2 +/− 26.7 vs. 30.1 +/− 15.6, p = 0.015).

### Hospice enrollment

Patients received PN for a median of 3.1 months (IQR: 1.6–5.5) before enrolling in hospice care (n = 40). Patients spent a median of 8.5 days (IQR: 4–22) in hospice prior to death. Five patients declined hospice care and two patients established home palliative care.

## Discussion

The use of PN in the context of end-of-life cancer care is a practice that necessitates improvement. As the treatment of cancer increasingly becomes management of a chronic disease process, appropriately identifying patients who will benefit from PN at the end-of-life is necessary. Therefore, our aim was to identify predictors of survival in order to further understand the benefit that patients may achieve with PN use. In addition, we focused on the complications and burden of PN therapy in the care of the patient. With respect to patient outcomes, we found a median survival of 4.7 months (IQR: 2.1–8), but also found a significant burden of hospital days at the end-of-life in patients pursuing use of PN for MBO.

Several international studies have similarly attempted to understand predictors of survival with the use of PN in the setting of advanced cancer. A retrospective review from Canada including 38 patients with advanced cancer receiving PN identified a median survival time of 162 days (5.4 months) and a Karnofsky performance score (KPS) greater than 50 at the time of PN initiation was associated with increased survival (9). In a similar study (N = 114) conducted in Poland, where gastric cancer was the most prevalent cancer type, a median survival of 89 days (2.9 months) was found (11). Sobocki et al. found that mild (male 10.0–14.0; female 10–12.0 g/dL), moderate (8.0–10.0 g/dL), and severe (6.5–8.0 g/dL) anemia and hypoalbuminemia (< 2.5 g/dL) were correlated with decreased survival. Though we did not identify any predictive laboratory values, we found that a prior diagnosis of appendiceal cancer was correlated with increased survival. Consistent with the above studies, we found a median survival of 142 days in a mixed cancer population among which the most common primary tumor sites were of appendiceal (35.3%) and colorectal (22.1%) origin.

With expanding interest in using prognostic factors to estimate survival probability in cancer patients receiving PN, Mariani, et al. used the Glasgow prognostic score (GPS), KPS, and primary tumor site to construct a nomogram capable of predicting 3-month and 6-month survival (12). While this nomogram was not validated in an external population, the general features of performance status and primary tumor site likely impact overall survival (13). In our data, the primary tumor site of appendiceal cancer predicted an improved survival. Future studies, primarily in the current era with novel available therapies will be required to elucidate disease and therapy related factors impacting survival among individuals with MBO.

The primary predictor of survival in our cohort was a prior diagnosis of appendiceal cancer. This increased survival can be explained by likely several mechanistic factors, the greatest being the general indolent nature of a mucinous adenocarcinoma of the appendix. While appendiceal cancer encompasses a number of different cancer subtypes that vary histologically and by disease outcomes, as opposed to other aggressive cancer types such as, colorectal and pancreatic cancers, the metastatic spread of disease is often limited to the abdomen becoming a space occupying tumor within the abdomen as opposed to spread to other organ systems (14). The poor response of appendiceal cancer to standard chemotherapies also likely reflects differences in host response to the tumor burden (15, 16). Since appendiceal cancers equated to the majority of cancer cases in this study, when evaluated separately from other cancer subtypes, median survival was 212 days in individuals with appendiceal cancer as opposed to 121 days in all other cancer subtypes.

Outside of individual patient survival, we also focused on outcomes of PN therapy in the setting of advanced cancer. Readmission after initiating PN is common. Kjeldsen et. al have found that nearly 80% of patients with incurable cancer receiving PN are readmitted into the hospital (17) We found an overall 30-day readmission rate of 52.2%. Older age was associated with a decreased likelihood of readmission within 30 days. Younger patients with metastatic cancer have been shown to experience higher readmission rates than older patients (18). Abdominal pain was the most common complication leading to hospital readmission in our study. Although symptom burden is highly variable, older patients with metastatic cancer generally report lower pain intensity (19). Age also affects clinician recommendations and individualized patient decisions (20). Older patients with metastatic disease experience higher levels of fatigue, emotional distress, loss of hope and pleasure, and independence. Furthermore, Cohen et. al found that older patients with metastatic pancreatic cancer are less likely to receive chemotherapy compared to younger patients (21). Older patients with metastatic cancer are also more likely to engage in early goals-of-care discussions, which have been associated with lower hospital readmission rates within 90-days (22). Though older age has been identified as an independent non-modifiable risk factor associated with less hospital readmission, it is a unidimensional measure and observed differences in hospital readmission are more likely multifactorial.

Specific to PN use, the CRBSI rate of 26% was high compared to the Canadian study that reported a line infection rate of 13% (9). Prior studies have identified risk factors for CRBSI, including using an intravenous port instead of a tunneled central line (23). Multiple technical explanations are plausible. Intravenous port catheters are more challenging to place, creating a higher threshold for removal (24) and small fluid reservoirs can build up if not flushed appropriately, leading to microbial growth within the catheter. Of note, intravenous ports were the most commonly used site for PN access in this cohort and likely contributed to the higher infection rate. Further assessment is needed in this cohort to identify the additional individual risk factors for developing CRBSI and minimize its impact on patient outcomes.

Lastly, we also demonstrated the burden of hospitalization on patients with a MBO on PN to include a median of 2 (IQR: 1–3) future readmissions, most commonly for worsening abdominal pain. Patients also spent a median of 29 (IQR: 19–44) days in the hospital after initial discharge on PN which accounts for nearly 21% of the patient’s survival time from PN initiation.

Quality of life on long-term HPN ranges from low to acceptable (25). Moreover, patients on HPN who depend on pain medications such as opiates or benzodiazepines experience a lower quality of life (26). Though hospital readmissions are a critical factor for defining patients’ quality of life on HPN, few evidence-based guidelines for clinical practice have been developed (27, 28). Future studies will have to consider the burden of hospitalizations in this specific patient population and its impact on quality of life.

At present, this is the largest single center study in the United States to describe the utilization of PN in the setting of an advanced cancer (n = 68). Comparable studies have been conducted in notable regions, such as Canada, Poland, and Italy, thereby prompting the need for pertinent comparisons across healthcare systems and patient demographics. Especially in the U.S. where delivery of PN can be fragmented, this large-scale collection allowed us to appropriately describe outcomes and burdens of PN therapy. However, given the heterogenous nature of the cancer subtypes included, specific predictors of survival outside of appendiceal cancer were not able to be determined and risk factors for hospital readmission also did not achieve statistical significance. Due to advancements and availability of chemotherapy and immunotherapy, identifying predictors of survival may prove to be a more complex analysis due to better outcomes with continuity of advanced treatment. Thus, gaining a comprehensive understanding of how innovative therapies influence treatment outcomes and overall survival becomes of paramount importance. The primary limitation of this study in addition to study size is the retrospective nature of the study which limited our ability to assess patient and family caregiver burden of PN utilization. Notably, the acquisition of hospice referral data relied upon the hospital’s electronic medical records. It is essential to acknowledge that certain instances of hospice referrals might have transpired without explicit documentation in the patients’ charts. When attempting to determine appropriateness of utilization, future studies will require prospective patient engagement and enrollment to determine patient and caregiver perspectives on the utilization of PN.

In summary, we show that patients with appendiceal cancers experienced greater survival than patients with non-appendiceal cancers. PN-associated complications occurred in many patients, notably, catheter related blood stream infections occurred in 18 (26%) patients. Patients survived an average of 142 days but also experienced an average of 2 readmissions and 29 days in the hospital after starting PN. Future work is needed to better characterize the patient experience on PN in cases of advanced cancer.

## Supplementary Material

Supplement 1

## Figures and Tables

**Table 1:** Overview of patient demographics for the study cohort. The table includes information on age, gender distribution, oncologic history, Eastern Cooperative Oncology Group (ECOG) scores, relevant laboratory values, treatment features, and survival.

Demographics	n = 68

Age, years, median (IQR)	57.5 (50.3–66)

Male sex, n (%)	30 (44.1)

White race, n (%)	42 (63.6)

Weight at PN initiation, kg, median (IQR)	66 (55.8–76.3)

Six-month weight loss before PN initiation, %, median (IQR)	8.4 (4.1–17.6)

Body mass index, median (IQR)	22.6 (20–26.2)

**Cancer features**	

Colorectal, n (%)	15 (22.1)

Gastric/gastroesophageal, n (%)	8 (11.8)

Gynecologic, n (%)	7 (10.3)
Fallopian tube: 2	
Ovarian: 3	
Uterine: 1	
Vaginal adenocarcinoma: 1	

Appendiceal, n (%)	24 (35.3)
Ex-goblet cell adenocarcinoma: 13	
Low-grade mucinous adenocarcinoma: 3	
High grade mucinous carcinoma: 1	
Metastatic appendiceal adenocarcinoma: 2	
Metastatic mucinous adenocarcinoma: 5	

Other, n (%)	14 (20.6)
Cholangiocarcinoma: 3	
Carcinoid: 1	
Laryngeal: 1	
Pancreatic: 1	
Peritoneal mesothelioma: 1	
Small bowel adenocarcinoma: 2	
Urothelial: 2	
Unknown primary: 3	

**ECOG, n (%)**	

0	6 (9.2)

29 (44.6)	
	
16 (24.6)	
	
14 (21.5)	
	
4	1 (1.5)

**Laboratory values at PN initiation**	

Hemoglobin, g/dL, median (IQR)	9.8 (8.8–11.7)

WBC, cells/10^3^uL, median (IQR)	7.8 (5.2–10.8)

Albumin, g/dL, median (IQR)	3.1 (2.8–3.5)

CRP mg/dL, median (IQR)	32 (9–76)

Nutrition Risk Index, median (IQR)	80.8 (73.3–85.4)

**Treatment features**	

History of exposure to chemotherapy, n (%)	68 (100)

Prior history of radiation therapy, n (%)	3 (4.4)

Prior surgical resection, n (%)	45 (66.2)

History of HIPEC, n (%)	17 (25)

Chemotherapy administered after MBO, n (%)	43 (66.2)

**Survival**	

Survival from PN start, months, median (IQR)	4.7 (2.1–8)

30-day survival, n (%)	58 (86.6)

90-day survival, n (%)	38 (57.6)

180-day survival, n (%)	23 (35.9)

**Table 2:** Cox regression time to event analysis to identify univariate predictors of overall survival. Patients with appendiceal cancer had statistically significant greater overall survival compared to patients with non-appendiceal cancers, demonstrating a reduced hazard ratio of 0.53 (95% CI: 0.29–0.92, p = .02) for death. No other baseline clinical predictors were significant to predict overall survival.

Demographics	HR	P-value
Age	1.01 (0.99–1.04)	0.390
Male sex	1.12 (0.67–1.87)	0.654
White race	0.9 (0.52–1.59)	0.7
Weight at PN initiation	1 (0.98–1.01)	0.686
Six-month weight loss before PN initiation	0.99 (0.96–1.02)	0.688
Body mass index	0.99 (0.95–1.04)	0.696
**Cancer features**		
Non-appendiceal cancer	Ref	
Appendiceal	**0.53 (0.29–0.92)**	**0.023**
ECOG 0–2	0.7 (0.39–1.32)	0.257
ECOG 3+	Ref	
**Laboratory values**		
Hemoglobin	0.92 (0.8–1.05)	0.217
WBC	0.97 (0.91–1.04)	0.386
Albumin	1.08 (0.69–1.68)	0.725
CRP	1 (0.99–1)	0.282
Nutrition Risk Index	1.02 (0.99–1.05)	0.215
**Treatment features**		
Chemotherapy administered after MBO	0.69 (0.41–1.19)	0.178

**Table 3:** Complications and hospital readmissions associated with PN use in advanced cancer patients. The median number of hospital readmissions and number of days spent in the hospital after starting PN were 2 readmissions (IQR: 1–3) and 29.5 days (IQR: 19.3–44), respectively. Fifty-one (75%) patients were subsequently admitted to the hospital for abdominal pain and 25 (36.8%) patients had hospitalizations for sepsis/bacteremia. Specific to PN utilization, 18 (26.5%) had a catheter related bloodstream infection (CRSBI). Five (7.3%) patients developed an upper extremity and 7 (10.3%) developed lower extremity deep vein thromboses (DVT). Twenty-three (33.8%) patients developed ascites and twenty-two (32.4%) patients developed pleural effusion.

Readmissions	n
Median number of readmissions	2 (Range: 0–10)
Median days spent in the hospital after initiation	29 (Range: 0–105)
**Complications**	**n = 68**
**Abdominal Pain**	51 (75.0%)
**Total Infections**	29 (42.6%)
Catheter Related Bloodstream Infection (CRBSI)	18 (26.0%)
Intraabdominal Perforation	4 (5.9%)
Biliary Drain Infection	1 (1.5%)
Gastrostomy Tube Infection	1 (1.5%)
Urinary Tract Infection	1 (1.5%)
Pulmonary Origin Infection	1 (1.5%)
No Clear Origin Infection	5 (7.4%)
**Sepsis**	25 (36.8%)
Line Related sepsis	12 (17.6%)
Non-line related sepsis	13 (19.1%)
**Deep Vein Thrombosis (DVT)**	12 (17.6%)
Upper Extremity	5 (7.3%)
Lower Extremity	7 (10.3%)
**Ascites**	23 (33.8%)
**Pleural Effusion**	22 (32.4%)

**Table 4:** Univariate analysis of predictors of 30-day readmission. The overall 30-day readmission rate was 52.2% (35/67). Older patients were less likely to be readmitted to the hospital (p = 0.037). There was no significant difference in hospital readmission rates related to gender, race, weight or weight loss > 10%, initial albumin value, malignancy type, or receipt of additional chemotherapy (p = NS).

	30-day readmission (n = 35)	No readmission (n = 32)	P-value
**Age, years, mean (SD)**	**55.5 (11)**	**60.9 (9.8)**	**0.037**
Male sex, n (%)	15 (42.9)	13 (40.6)	1
White race, n (%)	19 (55.9)	22 (68.8)	0.219
BMI, mean (SD)	23.6 (4.7)	23.8 (6.3)	0.841
Weight, lbs, mean (SD)	147.1 (38.8)	147.2 (39.5)	0.865
Weight loss > 10%, n (%)	15 (46.9)	13 (44.8)	1
Appendiceal cancer, n (%)	11 (31.4)	13 (40.6)	0.457
Albumin, g/dL, mean (SD)	3.1 (0.5)	3.1 (0.6)	0.792
Received additional chemo after PN, n (%)	20 (60.6)	21 (67.7)	0.609

## Data Availability

We performed a retrospective review over an 8-year period at our tertiary referral center identifying patients discharged on home parenteral nutrition for a malignant bowel obstruction.

## Abbreviations

ECOG: Performance Status Eastern Cooperative Oncology Group Performance Status
MBO: malignant bowel obstruction
PN: parenteral nutrition
TPN: total parenteral nutrition
