# Supplementary material for: Outcomes of parenteral nutrition in patients with advanced cancer and malignant bowel obstruction
Source: Res Sq. 2023 Nov 14:rs.3.rs-3455273. Preprint. [Version 1] doi: 10.21203/rs.3.rs-3455273/v1 (PMC10680932; doi:10.21203/rs.3.rs-3455273/v1)
Supplement: Supplement 1 [file NIHPPrs3455273v1-supplement-1.pdf]

Supplementary Table 1

Diagnosis codes utilized for capture of patients from electronic data warehouse.

**Bowel obstruction**	**560.8, 560.81, 560.89, 560.9**
	K56.50, K56.51, K56.52, K56.690, K56.691, K56.699, K56.600, K56.601, K56.609

AND (any of these):

**Intra-abdominal metastatic disease**	**197.4, 197.5, 197.6, 197.7, 197.8, 198.0, 198.1, 197.6, 197.7, 199.0**
	C78.4, C78.5, C78.6, C78.7, C78.89, C79.00, C79.11, C79.19, C80
**Malignant neoplasm of the stomach**	151.3, 151.4, 151.9, 151.8, 151.5, 151.6, 151.0, 151.1, 151.2
	C16.1, C16.2, C16.8, C16.9, C16.5, C16.6, C16, C16.4, C16.0
**Malignant neoplasm of the small intestine**	152.9, 152.8, 159.0
	C17.9, C17.8, C26.0
**Malignant neoplasm of the colon**	153.6, 153.1, 153.2, 153.3, 153.8, 153.9, 143.0, 153.7, 153.4
	C18.2, C18.4, C18.6, C18.7, C18.8, C18.9, C18, C18.3, C18.5, C18.0
**Malignant neoplasm of the rectosigmoid junction**	154.0,
	C19
**Malignant neoplasm of the rectum**	154.1, 154.8
	C20, C21.8
**Malignant neoplasm of the anus and anal canal**	154.3, 154
	C21.0,
**Malignant neoplasm of the pancreas**	157, 157.0, 157.1, 157.2, 157.9, 157.3, 157.8
	C25.0, 25.1, C25.2, C25.9, C25.3, C25.8, C25.7
**Malignant neoplasm of the bile ducts**	155.1, 156.1, 155, 156,
	C22.1, C24.0, C24.8
**Malignant neoplasm of the retroperitoneum and peritoneum**	158, 158.9, 158.8, 159, 159.9
	C48.2, C48.1, C48.8, C26.9
**Mesothelioma of the peritoneum**	158.8
	C45.1
**Malignant neoplasm of the appendix**	153.3
	C18.1

**Intra-abdominal metastatic disease**	**197.4, 197.5, 197.6, 197.7, 197.8, 198.0, 198.1, 197.6, 197.7, 199.0**
**Malignant neoplasm of the bladder**	188.9, 188.1, 188.5, 188.0, 188.3, 188.2, 188.4, 188.8, 198.1
	C67.9, C67.1, C67.5, C67.0, C67.2, C67.3, C67.4, C67.8, C66, C79.19
